# The Path of College Students’ Entrepreneurship Education Under Causal Attribution Theory From the Perspective of Entrepreneurial Psychology

**DOI:** 10.3389/fpsyg.2022.810615

**Published:** 2022-04-06

**Authors:** Changlin Wang, Qingquan Liu, Hongming Li, Yuanbing Liu

**Affiliations:** ^1^School of Economics and Management, Binzhou University, Shandong, China; ^2^College of Entrepreneurship, Jiaxing University, Jiaxing, China; ^3^Southampton Education School, University of Southampton, Southampton, United Kingdom; ^4^College of Teacher, Jiaxing University, Jiaxing, China

**Keywords:** entrepreneurship education, new venture management, entrepreneurial psychology, causal attribution theory, management strategy

## Abstract

The purpose of the study is to promote college students to actively respond to the national “Public Entrepreneurship and Mass Innovation” policies and calls, improve college students’ entrepreneurial enthusiasm and their entrepreneurial ability, and cultivate their good entrepreneurial psychological states. First, the relevant content of entrepreneurship psychology and causal attribution theory is displayed. Second, the questionnaire of college students’ entrepreneurship education is formulated and a questionnaire survey is conducted on University N based on the relevant content of entrepreneurship psychology. Subsequently, the management system of new venture A is taken as the research object to construct the management strategy of new ventures and simulate the implementation process. Finally, the questionnaire survey results of college students’ entrepreneurship education are analyzed and the corresponding entrepreneurship education path is formulated. Meanwhile, the implementation effect of the management strategy of new ventures is evaluated. After the questionnaire is sorted out, it is found that there are some problems in college students’ entrepreneurship education, such as weak awareness of entrepreneurship, insufficient publicity, outdated curriculum, and unqualified teachers. The reasons for these problems are the constraints of traditional concepts, insufficient attention, and incomplete system construction. Therefore, a plan is made for overall entrepreneurship education, the publicity of the concept of entrepreneurship education is strengthened, and the setting of entrepreneurship education curriculum and the ability of the teachers for entrepreneurship education are improved. Through the evaluation of the simulation implementation of a new enterprise management strategy, it is found that the new management strategy can achieve the expected effect. Therefore, this study provides some references for the development of college students’ entrepreneurship education and the management strategy of new ventures.

## Introduction

At present, national entrepreneurship education is still in its infancy. It is necessary to establish a perfect entrepreneurship education system and improve the popularity of entrepreneurship education in colleges (Jumana et al., 2020). College students encounter many problems in entrepreneurship, such as weak will, backward entrepreneurial consciousness, backward innovative thinking, weak sense of integrity, and weak psychological tolerance. These problems seriously affect the entrepreneurial behavior of college students (Cristian et al., 2020; Natalie and Eva, 2020), so they need to be improved. The improvement method is to comprehensively analyze the research on college students’ entrepreneurial psychology and then evaluate the results according to the knowledge of entrepreneurial psychology to solve the psychological problems of college students in the process of entrepreneurship. The ideological problems of college students are solved to guide students to improve their comprehensive quality, entrepreneurial enthusiasm and enthusiasm, and improve their innovative psychological state to improve the entrepreneurial effect of college students (Janeth et al., 2020). Next, start-ups are a new force in various industries. Its development has become the main industry development index, and its management strategy has also become the experimental focus of the main development of various industries. Hence, improving the management strategy of new enterprises is also the main task of the current social development. The research and improvement of the management strategy of start-ups can accelerate the development of start-ups, and comprehensively improve the management strategy of various industries (André, 2020; Hu and Meng, 2020; Ma et al., 2020; Rahul et al., 2020). Although the current optimization of college students’ entrepreneurship education and management strategy of start-ups is not ideal, many studies have provided technical support.

Scholars found that empowered leadership was positively correlated with followers’ feedback, and employees’ feedback was positively correlated with task performance, leaders and voice. They argued that employees’ feedback seeking mediated the positive relationship of empowered leadership and task performance, leaders and voice (Wu et al., 2019; Lubert and Gröpel, 2022), indicating that the challenges faced by foreign employees’ stem from assignments, unknown environments, language barriers, and cultural differences. Excessive pressure causes ideological and psychological burden to, and even lead to physical disorders. However, appropriate pressure can play a leading role in promoting the smooth progress of work (Chen, 2019); Steinsbekk et al. (2021) held that fragile self-esteem and admiration exhibit contradictory patterns of relationships with shyness and loneliness, while competition indicates low empathy. Wu (2019) stated that trust and profit can be regarded as the specific satisfaction of online entrepreneurial groups, especially trust, which is more worthy of attention in the further study of online entrepreneurship courses in social media. Wu and Song (2019) took MBA students from Tianjin University as samples to analyze the relationship of the dark triad, entrepreneurial self-efficacy, and EI. The results show that the dark triad positively predicted EI, ESE had a partial mediating effect on the dark triad and EI; Narcissism/psychopathy hurt ESE and EI; Narcissism/psychopathy had a non-linear impact on EI; Machiavellianism had a positive impact on ESE and EI; ESE had a mediating effect on the three members of the dark triad and EI. Wu W. et al. (2020) proposed a social business model for the disabled based on Eden’s mobile accessibility service platform. This social business model shows how information and communication technology can be combined with transportation service providers and government resources to meet the traffic needs of the disabled. Yuan and Wu (2020) believed that learners’ ability to work together and coordinate efforts in a team is becoming increasingly important for the success of any work and progress in knowledge. Wu Y. J. et al. (2020) found that the internal and external networks of BIs positively affect EGP, and exploratory and exploitative learning mediate the relationship between the two. When the incubator is in a highly dynamic environment, the internal network will more actively affect exploitative learning, while the external network will inhibit exploratory learning Chuenban et al. (2021) evaluated and analyzed the value of the CPC brand through Interbrand and Hirose models. Chen and Lin (2021) used systematic evaluation to comprehensively understand how to incorporate academic research into business intelligence to ensure the smooth implementation of a patient-centered medical system. Based on the theory of self-efficacy, Chen et al. (2020) and Feng and Chen (2020) constructed a model of the relationship between entrepreneurial enthusiasm and corporate psychology and behavior, put forward relevant, and proposed a promotion mechanism. Liu et al. (2021) introduced the concept of direct citation, co-citation, and bibliographic coupling of triangular citation, and defined three kinds of literature used in a triangular citation, namely original literature A, intermediate literature B, and subsequent literature C. Based on the uncertainty reduction theory, Deng et al. (2021) implemented a multi-level mediation model of the relationship between perceived environmental vitality and entrepreneurial team members’ innovation.

To sum up, first, the relevant contents of entrepreneurial psychology and causal attribution theory are displayed. In addition, according to the relevant contents of entrepreneurial psychology, the college students’ entrepreneurship education questionnaire is developed and distributed in University N. Then, with the new venture A management system as the research object, the new venture management strategy is constructed and its implementation process is simulated. Finally, the questionnaire survey results of college students’ entrepreneurship education are analyzed to formulate the corresponding path of entrepreneurship education. Next, the implementation effect of the business strategy of start-ups is evaluated. This exploration provides a reference for the optimization of college students’ entrepreneurship education system, and contributes to the comprehensive improvement of the management strategy of start-up enterprises.

## Research Process of Entrepreneurial Psychology and Causal Attribution Theory

### Theory of Entrepreneurial Psychology

**FIGURE 1:**
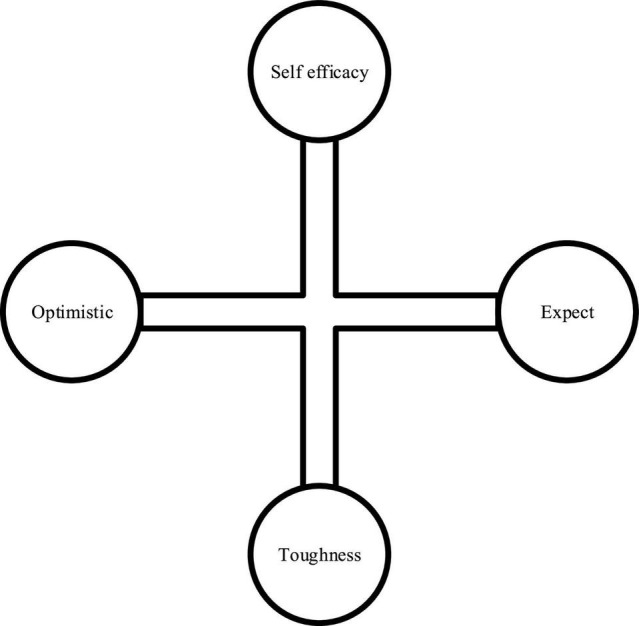
Dimensions of entrepreneurial psychology.

Entrepreneurial psychology is the vision of psychological capital theory proposed by Luthans, which is mainly reflected in entrepreneurs and entrepreneurial fields (Jena, 2020). Diane (2020) described that entrepreneurial psychology refers to the sum of psychological resources that can meet the emotional requirements of entrepreneurs in the entrepreneurial process and promote entrepreneurial success, which is reflected in the psychological state and constructive strength of entrepreneurs in entrepreneurial activities. João (2020) stated that entrepreneurial psychology refers to the integration of psychological resources that can meet emotional requirements in the entrepreneurial process and promote entrepreneurial success. The main measurement dimensions are shown in Figure 1.

Figure 1 shows that college students’ entrepreneurial self-efficacy can be defined as the ability to fully mobilize their resources and abilities to face challenging entrepreneurial environments and complete specific tasks. Optimism is that college students who want to start a business can view success and failure rationally, be good at self-evaluation objectively, summarize experience and lessons to the greatest extent, have a clear understanding of the responsibility relationship between themselves, groups, and the external environment, and can flexibly and calmly solve various events in entrepreneurship. Resilience can help entrepreneurial college students form strong social adaptability and a strong sense of social responsibility. Their socialization is faster than their peers, and they can grow rapidly into mature and independent entrepreneurial talents. Finally, the entrepreneurial goals and expectations set by entrepreneurial college students with hope are both realistic and challenging. They are also good at improving the possibility of achieving goals through self-guidance and careful planning. Hope is an “engine,” which can fully mobilize the individual’s positive initiative, thinking potential, and exploration spirit (Matthias and Sabine, 2020; Tingey et al., 2020).

### Causal Attribution Theory

**FIGURE 2:**
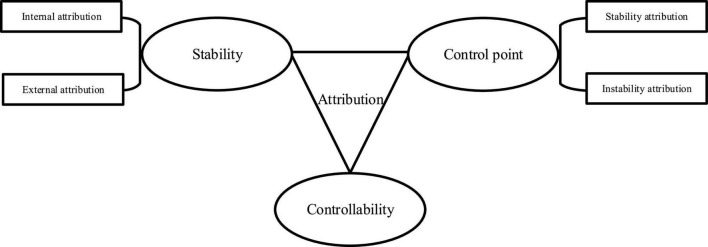
Distinguishing dimensions of the causal attribution theory.

The early causal attribution theory believes that the causes of behavior are composed of external environments and individual internal factors (Dai and Wang, 2020). With the emergence of the attribution control point theory, individuals are divided into internal control and external control (Xie and Wang, 2020). Individuals with internal control believe that the result of their ability to control life events is their ability or effort, while those with external control believe that they cannot control life events, and the result can only depend on luck or others (Wang et al., 2020). The three dimensions of attribution distinction in causal attribution are shown in Figure 2.

In Figure 2, the “control point” dimension of attribution can be divided into internal attribution and external attribution (Lee et al., 2020). The stability dimension of attribution can divide attribution into stable attribution and unstable attribution (Zhai et al., 2020). The controllability dimension refers to whether this reason is controllable or uncontrollable (Liu et al., 2020).

### Questionnaire Contents of College Students’ Entrepreneurship Education

Based on the theory of causal attribution and entrepreneurial psychology, this exploration studies the entrepreneurship education of college students and the management strategy of start-up enterprises. Hence, in the research process, it is necessary to conduct research and analysis according to the psychological investigation of students and the inquiry of managers of start-up enterprises. The questionnaire is mainly adopted to investigate students’ psychology, so the questionnaire mainly involves the following aspects: students’ ideas, motivation, factors of entrepreneurial success, entrepreneurial psychological quality, school entrepreneurial practice activities, frequency, measures to promote entrepreneurial activities, entrepreneurial curriculum content, entrepreneurial education teaching form, the composition of entrepreneurial education teachers, and teachers’ ability. The comprehensive criteria are causal attribution theory and entrepreneurial psychology.

### Methods and Process of Formulating Management Strategy of New Venture A

**FIGURE 3:**
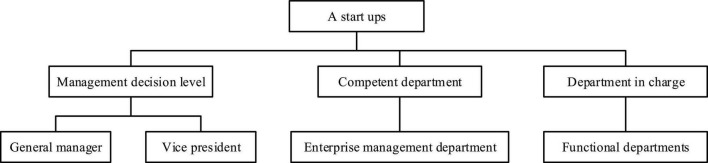
Internal organization settings of new venture A.

1. Main methods
  New ventures need to be strategic-oriented when formulating management strategies, and they take innovative R&D activities as the core to reduce innovation transaction costs. Attention should be paid to the cooperation among all functional departments such as marketing, procurement, production, and finance.
2. Organizational settings
  The management organization is mainly divided into three management departments, including decision-making departments, competent departments, and separate control. Organization settings are shown in Figure 3.
  The main responsibilities of management organizations are: the business decision-making departments are responsible for the implementation of management strategies and the realization of strategic objectives. The competent department is responsible for the overall management of management strategies. The separate control is responsible for the drafting and evaluation of the subordination strategy and the formulation of departmental plans.
3. Implementation plan
  The management strategy of new venture A is carried out in three stages, and the work plan is shown in Table 1.
4. The implementation effect of new venture A’s management strategyre A is evaluated by selecting corresponding indicators through the balanced scorecard. Then, from the four perspectives of learning and growth, business process, customer and finance, the effect of management process system optimization is evaluated. Table 2 is the evaluation index of the implementation of the business strategy of new venture A. The comprehensive performance of enterprises in all aspects is analyzed, which combines the research on managers’ psychology, and analyzes the current situation of management strategies of start-ups according to the causal attribution theory and the basic concept of entrepreneurial psychology. Moreover, its development characteristics are comprehensively sorted to analyze the development direction and purpose of the new entrepreneurship management strategy. The calculation equation of labor productivity is:

**TABLE 1:** Implementation plan of the management strategy of new venture A.

Stages	Implementation time	Implementation content
1. Start-up phase	January 2020–February 2020	1. Establish the project team. 2. Hold the kick-off meeting
2. Implementation phase	February 2020–November 2020	Review, optimize, approve and release the document of the management strategy.
3. Evaluation stage	December 2020	Summarize and evaluate the documents of the management strategy, and formulate a continuous improvement mechanism based on the improvement.

**TABLE 2:** Evaluation indexes of implementation.

Evaluation dimensions	Indexes	Method and purpose
Learning and growth index	Labor productivity	Analyze the development potential of business managers through psychology.
	Average annual training time for staff	
	Number of staff-improving proposals	
Business process index	Number of new products open listing	Psychological analysis of business personnel development potential of enterprises.
	Number of national patents obtained	
	Product qualification rate	
Customer index	Market share	Psychological analysis of the development potential of enterprise market personnel.
	Increase rate of new customers	
	Customer satisfaction	
Financial index	Operating income	Analyze the development potential of enterprise service personnel through psychology.
	Net profit	


(1) $$\mathrm{labour}\,\mathrm{productivity}=\frac{\mathrm{output}\,\mathrm{value}}{\mathrm{total}\,\mathrm{number}\,\mathrm{of}\,\mathrm{employees}}$$

The calculation equation between the average annual training of employees is:


annual⁢training⁢time⁢of⁢employees

(2) $$=\frac{\mathrm{sum}\,\mathrm{of}\,\mathrm{training}\,\mathrm{time}\,\mathrm{for}\,\mathrm{employees}}{\mathrm{number}\,\mathrm{of}\,\mathrm{participants}}\times 100\%$$

The number of employee-improving proposals is the total number of employee-improving proposals in 2020. The number of new product development listings is the total number of new product development listings in 2020. The number of national patents can be evaluated by the total number of national patents obtained by new venture A. The calculation equation of product qualification rate is:


product⁢qualification⁢rate=

(3) number⁢of⁢qualified⁢products⁢in⁢ 2020total⁢number⁢of⁢products⁢in⁢ 2020×100%

The calculation equation of market share is:


(4) $$\mathrm{market}\,\mathrm{share}=\frac{\mathrm{product}\,\mathrm{sales}}{\mathrm{sales}\,\mathrm{of}\,\mathrm{products}\,\mathrm{in}\,\mathrm{the}\,\mathrm{industry}}\times 100\%$$

The calculation equation of the increase rate of new customers is:


growth⁢rate⁢of⁢new⁢customers

(5) $$=\frac{\mathrm{number}\,\mathrm{of}\,\mathrm{new}\,\mathrm{customers}\,\mathrm{in}\,\mathrm{current}\,\mathrm{period}}{\mathrm{number}\,\mathrm{of}\,\mathrm{customers}\,\mathrm{in}\,\mathrm{the}\,\mathrm{previous}\,\mathrm{period}}\times 100\%$$

Customer satisfaction is analyzed mainly through the customer satisfaction to goods or services of new venture A. The calculation equation of operating income is:


business⁢income=main⁢business⁢income

(6) +other⁢business⁢income

The calculation equation of the net profit is:


(7) net⁢profit=total⁢profit×(1-income⁢tax⁢rate)

**FIGURE 4:**
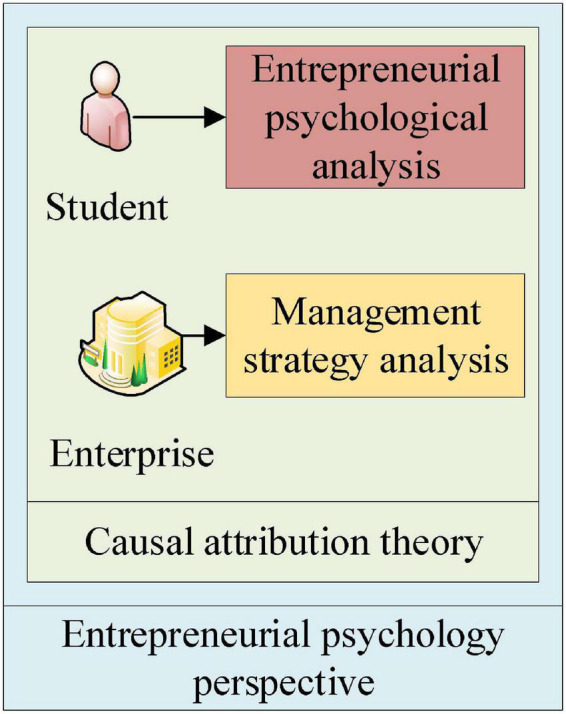
The research idea of entrepreneurship education for college students and management strategy research of start-up enterprises.

By calculating the above equation, a comprehensive analysis of start-ups can be made in many aspects to study the development of management strategies of start-ups deeply. Figure 4 shows the basic idea of studying college students’ entrepreneurship education and start-up enterprise management strategy based on causal attribution theory from the perspective of entrepreneurial psychology.

Figure 4 shows that the basic research idea is to conduct a comprehensive study on college students’ entrepreneurship education path and start-up enterprise management strategy based on causal attribution theory from the perspective of entrepreneurial psychology.

## Data Analysis

### Data Analysis of the Questionnaire on College Students’ Entrepreneurship Education

**FIGURE 5:**
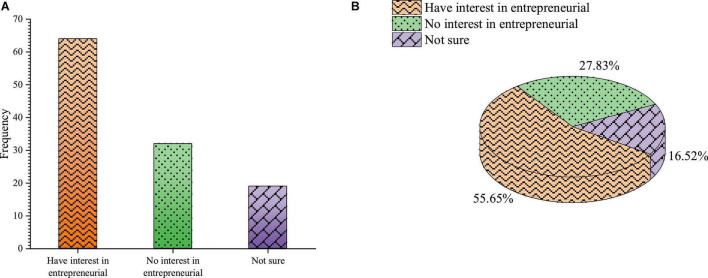
College students’ attitude toward entrepreneurial interest **(A)** frequency **(B)** percentage.

1. College students’ cognition of entrepreneurship education College students’ attitude toward entrepreneurial interest is shown in Figure 5.

**FIGURE 6:**
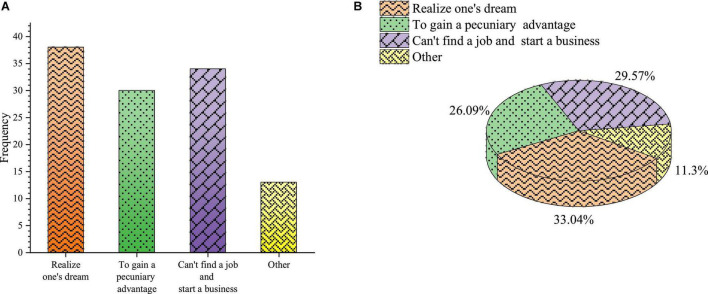
College students’ entrepreneurial motivation **(A)** frequency; **(B)** percentage.

The survey results of students’ interest in entrepreneurship show that only a few students have little interest in entrepreneurship. Figure 5 shows that 55.65% of students have entrepreneurial interest, only 27.83% have no entrepreneurial interest, and 16.52% are uncertain. It shows that most college students are very interested in entrepreneurship. However, the requirements of entrepreneurship for students are not simple. Therefore, it is very important to investigate whether college students have entrepreneurial ideas and entrepreneurial motivation. Figure 6 shows the investigation results on college students’ entrepreneurial motivation.

In Figure 6, the survey results of entrepreneurial motivation show that 33.04% of students choose to realize their entrepreneurial dreams, 26.09% choose to make money, 29.57% start a business because they cannot find a job, and 11.3% choose other options. The proportion of students who choose to realize their dreams is the highest of all options. Multiple students are motivated by scientific entrepreneurship, but most of them want to make money. Therefore, in the initial entrepreneurship motivation education, schools should correctly guide college students to make them have correct and scientific entrepreneurship motivation and value orientation under the role of entrepreneurship education.

2. Campus atmosphere

**FIGURE 7:**
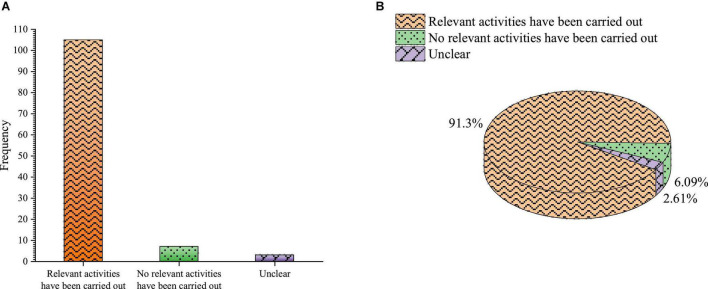
Whether the university has carried out entrepreneurship education-related activities **(A)** frequency **(B)** percentage.

Figure 7 shows whether universities have carried out entrepreneurial education-related activities.

Figure 7 shows that 91.3% of students choose to participate in entrepreneurship education lectures, competitions and other activities. 6.09% are not very clear about the activities related to entrepreneurship education carried out by the school, and only 2.61% choose not to participate in the activities related to entrepreneurship education held by any unit. It reveals that most students know that colleges have set up activities related to entrepreneurship education, which reflects the publicity of entrepreneurship education activities and the importance of entrepreneurship education.

**FIGURE 8:**
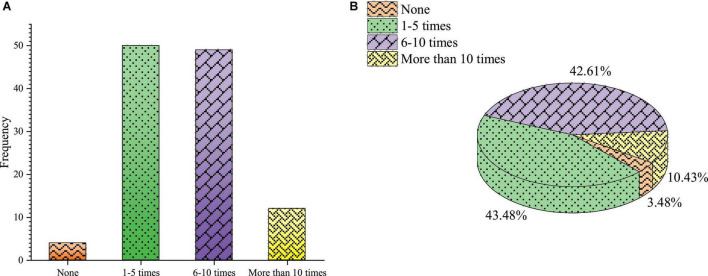
Entrepreneurship education-related activities opened by the university **(A)** frequency **(B)** percentage.

The frequency of school entrepreneurship education-related activities per semester is shown in Figure 8.

Figure 8 shows that in the frequency of entrepreneurship education-related activities held by the school every semester, the number of students who choose 6–10 times and 1–5 times is more, accounting for 42.61 and 43.48%, respectively. 10.43% students choose 10 times and 3.48% students choose 0 times. The entrepreneurial activities held by the school are concentrated at 1–10 times per semester, and the frequency of entrepreneurial activities is reasonable. However, a few students know nothing about it and do not participate in it. It shows that colleges should continue to strengthen the publicity of entrepreneurial activities.

3. Entrepreneurship curriculum

**FIGURE 9:**
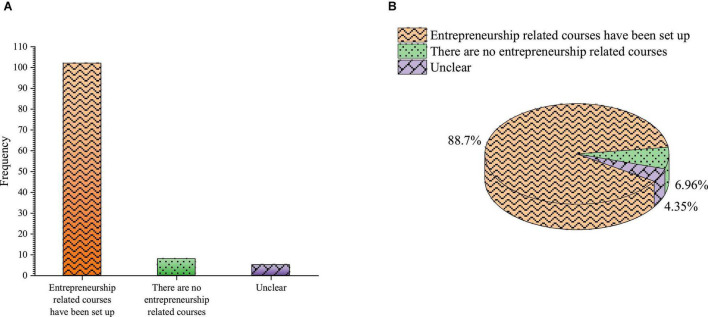
Whether the university opens entrepreneurship curriculum **(A)** frequency **(B)** percentage.

Whether the university opens the entrepreneurship curriculum is shown in Figure 9.

Figure 9 shows that 88.7% of students choose to study courses related to entrepreneurship, 6.96% of students hold that the school does not offer entrepreneurship-related courses, and 4.35% of students do not know whether to offer entrepreneurship-related courses. It shows that the school has set up entrepreneurship-related courses, and only a few students do not know that. However, schools need to continue to strengthen the publicity of entrepreneurship education courses to popularize entrepreneurship education courses and enable students to enjoy equal teaching rights.

**FIGURE 10:**
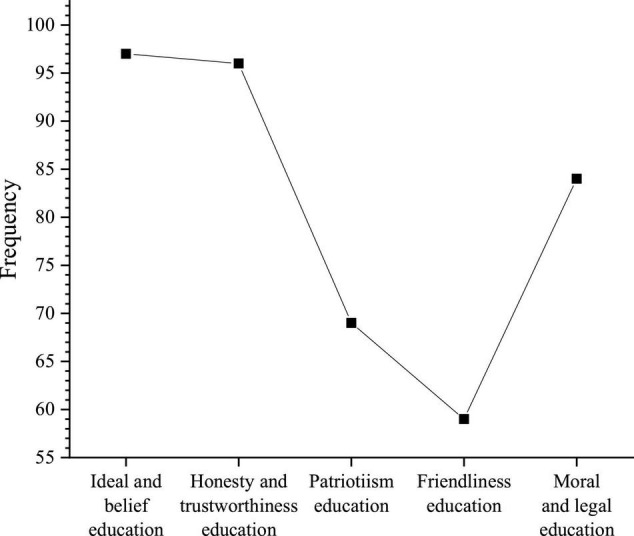
Contents of entrepreneurship curriculum.

The content of the entrepreneurship curriculum is shown in Figure 10.

Figure 10 shows that the majority of students choose ideals and beliefs and honesty and trustworthiness, which are 97 and 96 students, respectively. The number of students who choose moral and legal education, patriotism, and goodwill is 69, 59, and 84, and the number of students who choose nothing is 22. Ideal and faith, patriotism, friendship, integrity, morals, and the legal system are the basic contents of ideological and political education. When students’ entrepreneurship education is carried out, more attention should be paid to the strengthening of the above contents. In the university’s entrepreneurship curriculum, ideal faith and honesty account for a large proportion. These two aspects are also the most important aspects of entrepreneurship education. Meantime, universities should also pay attention to the cultivation of students’ moral and legal systems, patriotism, and friendship.

4. The teachers for entrepreneurship education

**FIGURE 11:**
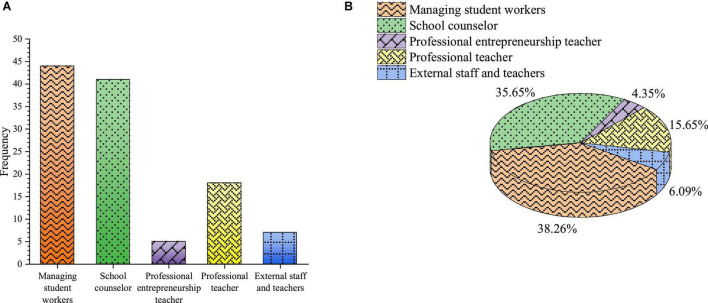
Teachers for entrepreneurship education **(A)** frequency **(B)** percentage.

The teachers for entrepreneurship education are shown in Figure 11.

Figure 11 shows that the percentages of students who choose to manage student workers and counselors are high in the survey, and they are 38.26 and 35.65 %, respectively. The percentage of students who choose professional teachers is 15.65%, the percentage of students who choose external staff is 6.09%, and that of students who choose full-time entrepreneurial teachers is 4.35%. The composition of teachers for ideological and political education is complex. Teachers and counselors who manage student work are the backbones of entrepreneurship education. Full-time entrepreneurship teachers can better guide students to establish entrepreneurial values, but the size of full-time teachers is small.

**FIGURE 12:**
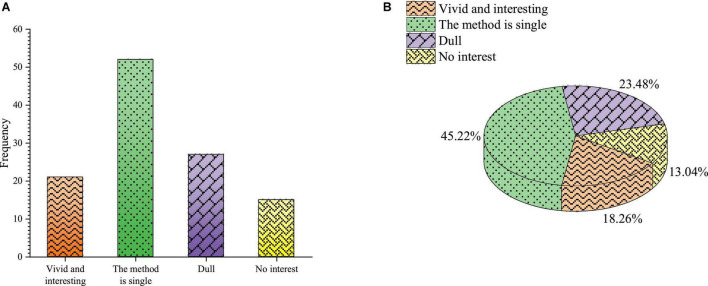
Students’ feelings in the entrepreneurial education **(A)** frequency **(B)** percentage.

The students’ feelings about entrepreneurship classroom teaching are shown in Figure 12.

Figure 12 shows that 45.22% of the students choose the single method of entrepreneurship education, 23.48% of the students think that the classroom teaching of entrepreneurship education is boring, and 18.26 and 13.04% of the students think it is lively and interesting. This shows that students generally believe that the classroom teaching of entrepreneurship education is boring, the teaching method is single, and the courses taught by teachers are only in the form of classroom teaching. Students prefer teachers to develop various forms of entrepreneurship knowledge. Teachers need to be further strengthened in professional skills and teaching abilities.

### Data Analysis Before and After the Implementation of the Management Strategy of New Ventures

**FIGURE 13:**
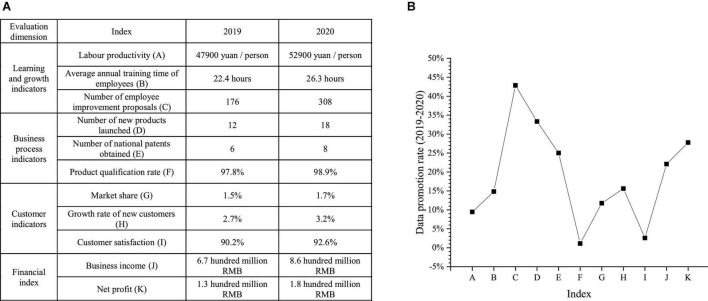
Comparison of index data for 2020 and 2017 **(A)** specific data **(B)** data enhancement rate.

The balanced scorecard is used to evaluate the effect of management process system optimization from the perspectives of learning and growth, business process, customers, and finance, and to analyze the comparison of index data between 2020 and 2019, as shown in Figure 13.

Figure 13 shows that in 2020, the number of new products listed and the number of national patents obtained by new venture A increase significantly compared with that in 2019, the labor productivity increases by 9.45% compared with that in 2019, and the qualified rate of enterprise products is as high as 98.9%. Meanwhile, the market share, the increase rate of new customers, and customer satisfaction increase differently. In 2020, new venture A’s operating income and net profit hit a record high.

## Discussion

This exploration is to study the path of college students’ entrepreneurship education and the management strategy of start-up enterprises based on causal attribution theory from the perspective of entrepreneurial psychology. The purpose is to solve the psychological problems faced by college students in the process of entrepreneurship and the specific problems faced by the managers of start-up enterprises in the process of management.

### Path of College Students’ Entrepreneurship Education

First, the questionnaire survey results on the current situation of college students’ entrepreneurship education show that colleges should strengthen the effectiveness of entrepreneurship education. The most important path is to plan the overall layout of entrepreneurship, improve students’ entrepreneurial awareness, strengthen entrepreneurship education and publicity, create a strong entrepreneurial atmosphere, improve entrepreneurship courses, innovate development models and improve entrepreneurial teachers’ abilities. It is essential to lay a solid foundation for students’ entrepreneurship education and improve their psychological quality through comprehensive psychological education to strengthen their entrepreneurship ability.

1. It is necessary to integrate the overall planning of entrepreneurship education and improve students’ entrepreneurship awareness. Entrepreneurship education in colleges should aim at the all-round development of students, and respond to national rules, regulations and policies.
2. It is necessary to strengthen the concept of entrepreneurship education and create a strong campus entrepreneurship atmosphere. Strengthening the publicity of entrepreneurship education through the campus cultural atmosphere can make entrepreneurship education cover all aspects of students’ life. Besides, it can help students have the correct concept, change the incorrect entrepreneurial concept, and promote the development of entrepreneurship education in colleges.
3. It is necessary to improve the curriculum design of entrepreneurship education and innovate the new model of entrepreneurship education. In the curriculum and teaching of entrepreneurship education, colleges can fully combine the ideological and political education standards, formulate student training plans, and innovate the current entrepreneurship education model to adapt to social development and national talent needs, and promote the development of entrepreneurship education for college students.
4. It is necessary to improve teachers’ entrepreneurial education ability and ensure students’ entrepreneurial reserve. It is also necessary to form a pattern of all staff education in entrepreneurship education for college students. Schools should have professional teachers with rich theoretical and practical experience and constantly improve teachers’ ability to ensure the realization of college students’ entrepreneurship education objectives.

Students’ entrepreneurial ability can be comprehensively improved by improving the educational system and methods. The most important thing is to solve the psychological problems students face when starting a business to reduce the entrepreneurial resistance of students themselves. Compared with Cui et al. (2021), this exploration has a clearer research direction, and the factors contained in students’ psychological research are more comprehensive.

### Management Strategy of New Ventures

Next, according to the characteristics of new venture A, it is found that it has set up a special team, system and competent / competent department, organized and constructed a relatively perfect and standardized management process system, solved the current management problems of the enterprise, and formed a good development trend and the trend of continuous improvement. Besides, the research shows that the following work in the management of start-ups can continuously improve the improvement of their management strategies and comprehensively improve the development status of enterprises:

1. Continuous promotion of innovation. According to the characteristics, development status and existing problems of the enterprise, the management process system is designed to ensure that the internal management system of the enterprise can adapt to the strategy and development direction. Scientific and perfect management process system is an important embodiment of improved management ability. Through the management process system, the input of the enterprise is transformed into the expected output, so that the enterprise can obtain the endogenous driving force of sustainable innovation.
2. Efficiency improvement. After the overstaffed management links are simplified, the weak management links are refined, reasonable process direction and management requirements are formulated, and an efficient management process system is established to avoid mutual prevarication due to unclear responsibilities and interfaces. It is necessary to strengthen cross-departmental communication and cooperation, reduce repeated and disorderly management, greatly improve work efficiency and reasonably allocate internal resources.
3. Product quality improvement. Product research and development starts from customer needs. The management process system is designed with research and development activities as the core. Its ultimate goal is to provide customers with high-quality products and better services and enhance the corporate image. The increase of process and system key control points has effectively supervised the quality of products from research and development to production and then to customers, and guaranteed the product quality of the enterprise.
4. Improvement of economic benefits. In the management process system, some redundant and complex links are simplified. Due to the improvement of work efficiency, the enterprise’s management cost and labor cost are saved. Moreover, it shortens the product development cycle and delivery time, and reduces the capital cost. The listing of new products brings new profit growth points. This series of changes will eventually improve the economic benefits of enterprises.

This exploration is to comprehensively analyze the current development status of start-ups and study the development direction and concept of start-ups to provide them with comprehensive development power and promote the development of the whole society. Compared with the research of Du and Kim (2021), the research and analysis factors of the management strategy of start-ups here are more extensive, and the research content is more novel, which plays a greater role in the development of start-ups.

## Conclusion

Based on the full investigation of college students’ entrepreneurship concept and entrepreneurship education teaching content, this exploration is to analyze the current situation of college students’ entrepreneurship education, and put forward the countermeasures to strengthen entrepreneurship education. The purpose is to improve the level and quality of college students’ entrepreneurship education, cultivate excellent college students who meet the needs of the country and society, and make them have the innovative and entrepreneurial spirit, entrepreneurial ability and quality. Moreover, relevant theoretical knowledge such as entrepreneurial psychology and attribution is used to design its management process system. Then, an enterprise integrated management process system that can be continuously improved is constructed through the overall strategic conception and optimization of new venture A in comprehensive enterprise management. It plays an important role in the overall management optimization of start-ups. Due to the complexity of the research object and the limitation of time, two points need to be improved. (1) In the study of the entrepreneurial path of college students, the objects of the questionnaire are only the students of University N. As a college of Arts and Sciences, University N has low reference value for the research of entrepreneurship education for science college students. (2) The discussion of the business strategy of new venture A does not include the internal environment of start-up enterprise, but only the research on the overall management of start-up enterprise. It is necessary to strengthen the in-depth research on the investigation content of start-up enterprise. In-depth research will be conducted according to these two shortcomings in the future.

## Data Availability Statement

The raw data supporting the conclusions of this article will be made available by the authors, without undue reservation.

## Ethics Statement

The studies involving human participants were reviewed and approved by the Jiaxing University Ethics Committee. The patients/participants provided their written informed consent to participate in this study. Written informed consent was obtained from the individual(s) for the publication of any potentially identifiable images or data included in this article.

## Author Contributions

All authors listed have made a substantial, direct, and intellectual contribution to the work, and approved it for publication.

## Conflict of Interest

The authors declare that the research was conducted in the absence of any commercial or financial relationships that could be construed as a potential conflict of interest.

## Publisher’s Note

All claims expressed in this article are solely those of the authors and do not necessarily represent those of their affiliated organizations, or those of the publisher, the editors and the reviewers. Any product that may be evaluated in this article, or claim that may be made by its manufacturer, is not guaranteed or endorsed by the publisher.
